# Snakehead Consumption Enhances Wound Healing? From Tradition to Modern Clinical Practice: A Prospective Randomized Controlled Trial

**DOI:** 10.1155/2018/3032790

**Published:** 2018-11-14

**Authors:** Nik Amin Sahid, Firdaus Hayati, Challa Venkata Rao, Rosnelifaizur Ramely, Ikhwan Sani, Andee Dzulkarnaen, Zaidi Zakaria, Syed Hassan, Arman Zahari, Aishath Azna Ali

**Affiliations:** ^1^Surgery Department, Faculty of Medicine and Health Sciences, Universiti Malaysia Sabah, 88800 Kota Kinabalu, Sabah, Malaysia; ^2^Surgery Department, School of Medical Sciences, Hospital Universiti Sains Malaysia, 16150 Kubang Kerian, Kelantan, Malaysia; ^3^Plastic & Reconstructive Surgery Department, School of Medical Sciences, Hospital Universiti Sains Malaysia, 16150, Kubang Kerian, Kelantan, Malaysia; ^4^Surgery Department, Indra Gandhi Memorial Hospital, Kanbaa Aisa Rani Higun, Malé, Maldives

## Abstract

**Background:**

Snakehead fish (*Channa striatus)* is a fresh water fish indigenous to many Asia countries and believed to have medical value. Studies showed that it contains all the essential amino acids and fatty acids able to accelerate wound healing and it has antinociceptive effect. However, little human study has been done to assess the effectiveness of* Channa striatus *in wound healing. A prospective RCT has been conducted on the effect of* Channa striatus *spray versus placebo on clean wound to assess its pain control effect and cosmetic outcome.

**Methodology:**

One hundred and two patients (102) underwent clean elective surgery; postoperatively they were randomized into two group. One group received* Channa striatus *extract spray (n=51) another group received placebo (n=51) on daily basis for 2 weeks. They were followed up on 2^nd^, 4^th^, and 6^th^ weeks. Pain control effect was assessed based on Visual Analog Pain Score (VAPS) and cosmetic outcome based on Visual Analog Cosmetic Scale (VACS), Wound Evaluation Scale (WES), and Vancouver Scar Scale (VSS).

**Result:**

The patient treated with* Channa striatus *spray displayed a better outcome in terms of pain control compared to placebo. During analysis using repeated measure ANOVA, there was significant difference of patient's pain score based on VAPS between* Channa striatus *spray and placebo (F-stat (df) = 4.80 (2), p-value = 0.010). For cosmetic outcome it showed a better result in* Channa striatus* spray group for all the 3-scoring system, VACS, (F-stat (df) = 2.68 (2) , p-value <0.001), WES (F-stat (df) = 3.09 (2), p-value = 0.048), and VSS (F-stat (df) = 1.72 (2) , p-value = 0.011).

**Conclusion:**

Our study suggest that application of* Channa striatus* extract spray on clean wound has shown a significant better pain score result and cosmetic outcome on week 2, week 4, and week 6 comparatively with placebo.

## 1. Introduction

Snakehead fish (*Channa striatus) *flesh is claimed to be rejuvenating, particularly in recuperation from serious illness and in a postnatal diet. In Malaysia, it has always been a strong belief that* Channa striatus* enhances wound healing and is a very powerful tool for recovery of health and injury. Since 1931 there has been in Malaysian literature discussion about wound treatment using* Channa striatus*. Several studies showed that it contained all the essential amino acids and fatty acids (Table 1) which uniquely are capable of accelerating the wound healing [1, 2].

Despite the wide-spread uses of this fish for medicinal purposes, very little studies to establish the scientific basis for it claimed wound healing effects. In an animal study,* Channa striatus* extract has been shown to increase the tensile strength of the surgically stitched wound. It also has been formulated into aerosol/spray for drug delivery system to wound and burn treatment [3, 4]. Evaluation of the film properties from concentrate of aerosol had been done in other study [5]. But the effect on human has not been done yet. Therefore the effects of* Channa striatus* extract in aerosol form on clean surgical wounds are evaluated in this study.

### 1.1. Snakehead Fish (*Channa striatus*)

*Channa striatus* is a fresh water species which is also known as snake head fish or known as Haruan in Malay. It belongs to Channidae family and it is carnivorous fish.

### 1.2. Traditional Belief on* Channa striatus*

Traditionally Chinese and Malay community believes that eating* Channa striatus* during postpartum period is enhancing wound healing. It also believe that* Channa striatus* acts as energy booster meal based on a study done among Chinese respondents in a Kuala Lumpur maternity hospital involved questions on the consumption of* Channa striatus* [6].

### 1.3. Nutrition Composition of* Channa striatus*

Study found that* Channa striatus* extract is rich in amino acids, a nonessential amino acid which is glutamic acids, arginine, and aspartic acid [7]. Others were listed in the table below.

### 1.4. Composition of Channa striatus Spray

Snakehead fish (*Channa striatus)* water extract has been formulated in an aerosol system which can produce a film for wound dressing. It was manufactured by Skin Fix Company. Snakehead fish (*Channa striatus) *spray has been evaluated for the possibility of causing irritation reaction or toxic response; however from three experiments carried out to evaluate the safety of Snakehead fish (*Channa striatus)* spray which are Primary Skin Irritation test, Intracutaneous test, and Systemic Injection test, the result shows that Snakehead fish (*Channa striatus)* spray gave no significant responses to all the above tests [5, 8]. In 2011, Febriyenti has formulated an aerosol concentrate containing a mixture of Snakehead fish (*Channa striatus)* extract and a film-forming polymer. The concentrate when sprayed on the wound formed a thin layer of dressing and the added Snakehead fish (*Channa striatus)* extract proved to enhance the healing process as proven by Baie and Sheikh who studied the wound healing effect of* C. striatus* on* Sprague-Dawley* rats [9].

### 1.5. *Channa striatus* in as Antimicrobial

As a part of the wound healing process, antimicrobial activity is equally important. The antimicrobial properties of the skin and intestinal mucus of different* Channa *sp*., namely, C. striatus, Channa micropeltes, Channa marulius, Channa punctatus, *and* Channa gachua* have been studied by CARE research team. The investigation showed a broad spectrum of antibacterial activity of skin mucus against* Aeromonas hydrophila*,* Pseudomonas aeruginosa,* and* Vibrio anguillarum* [10].

### 1.6. Antinociceptive Properties Snakehead Fish (*Channa striatus)*

The analgesic or antinociceptive effect were being studied by a few researchers. For instance, Mat Jais et al. (1997) investigated the antinociceptive effects in mice with a view to establishing the scientific basis of pain-relieving activities where the study showed that both the fillet and mucus of* Channa striatus* were found to exhibit a concentration dependent antinociceptive activity [11]. There are evidences for arachidonic acid of haruan enhancing the activity of other antinociceptive agents such as morphine [11].

### 1.7. Rationale of Study

Clean surgery procedure is one of major bulks of general surgery work load. It occupies almost 40% of all elective case. Postoperative pain and cosmetic outcome are of major concern for the patient and potentially debilitating. Previous animal study has proven that Snakehead fish (*Channa striatus)* extract has improved tissue healing. However there are only few human studies done regarding the effect of Snakehead fish (*Channa striatus)* spray on cosmetic outcome and pain control. It is clinically useful if we can identify the effectiveness of Snakehead fish (*Channa striatus)* extract spray on clean wound, which can be potentially extended to clean contaminated wound in future.

## 2. Methodology

### 2.1. Study Subject/Source Population

This is a randomized, prospective, clinical study to evaluate the effect of topical administered Snakehead fish (*Channa striatus)* extract in aerosol/spray form with the effect of placebo (spray without Snakehead extract). Subjects were recruited from Clinic of General Surgery (SOPD), Universiti Sains Malaysia (convenience based sampling). Patients were scheduled for an elective operation with clean incisional wounds that were primarily sutured. They were subjected to face to face interview to enquire about their suitability of the study. Eligible subjects consented to participate were randomly assigned to one of the two groups:

  Group 1: subjects received Snakehead fish (*Channa striatus)* spray.
  Group 2: subjects received placebo spray.

Subject must fulfill each of the following criteria.

*Inclusion Criteria*

- Age ≥ 18 and ≤ 50 years
- Subject who has given written informed consent to participate in the study and understand the nature of the study

*Exclusion Criteria*

- Taking any form of herbal extract in the last 3 months before study entry and during the study period
- History of drug or alcohol abuse
- Patient taking warfarin or heparin
- Clinical relevant cardiovascular, gastrointestinal, hepatic, neurologic, endocrine, hematologic, connective tissue disease or other major systemic diseases that would influence the interpretation of results
- Patients with medical disorder requiring steroid or immunosuppressive therapy with delay wound healing
- Patient with chronic cough or other condition which may cause a rise in intra-abdominal pressure
- Presence of any congenital anterior abdominal wall defects
- Patient with evidence of secondary infection after treatment
- Mental condition rendering the subject unable to understand the nature, scope, and possible consequences of the study
- Evidence of uncooperative attitude, including poor compliance including inability to attend follow-up visit

### 2.2. Method of Assigning Subjects to Treatment and Placebo Groups

Subject eligibility was established before treatment randomization. Subjects' number was allocated strictly sequentially, as subjects were eligible for randomization. A randomization method using randomization software is at www.randomization.com. Number that has been chosen by the software will determine whether the patient will get either treatment A or treatment B. None of the investigators knows the randomization scheme.

### 2.3. Blinding and Procedures for Breaking the Blind

This was double blinded study and once a subject has been randomized, the study treatment that they received was not be known by both the subject and the investigator.

### 2.4. Patient's Withdrawal

The investigator may cease study treatment and withdrew the subject or the subject may withdraw herself from participation in the study at any time. The reason for the withdrawal of a patient will be recorded in the case report form. Subject were followed-up for a minimum of 42 days (6 weeks) following the last dose of study drug.

Possible reasons for patient withdrawal include the following:

- The need to take medication may interfere with study measurements.
- Patient experiences an intolerable/unacceptable adverse event.
- Patient exhibits noncompliance with the protocol.
- Patient unwilling to proceed and/or consent is withdrawn.
- Investigator withdraws patient for reasons unrelated to the study drug (e.g., undercurrent illness)

## 3. Materials

### 3.1. Investigational Products

The topical administered Snakehead fish (Channa striatus) spray and placebo (spray without Snakehead fish (Channa striatus) extract) were prepared in GMP Laboratory, School of Pharmacy Universiti Sains Malaysia. The preparation of the concentrates followed the method that was described in detail in previous study [5].

### 3.2. Doses and Treatment Regimens

The treatment group were sprayed with Snakehead fish (*Channa striatus)* spray once a day while the placebo group were sprayed with placebo spray (spray without Snakehead fish) (*Channa striatus) *extract once a day.

### 3.3. Ethical Clearance

Ethical clearance has been obtained from Human Research Ethical Committee USM (HREC), USM/JEPeM/1403124

### 3.4. Data Collection Procedure

Basic demographic data were collected from the patient and surgical procedure, indication, and method of wound closure were gathered. The wound assessment was performed by clinical assessment using Visual Analog Cosmetic Scale (VACS), Wound Evaluation Scale (WES), and Vancouver Scar Scale (VSS) by the investigators and also by the patient using Visual Analog Pain Score (VAPS). A photo of the wound was taken serially at every visit and assess by two independent investigators using VACS, WES, and VSS. Snakehead fish (*Channa striatus)* spray or placebo spray were used to protect the wound after postoperative wound inspection. All subjects were instructed to take normal diet during the study period and were not be allowed to take any other herbal products orally or consume* C. striatus*. The cosmetic assessments of the wound were done by the investigator who is part of the Clinical Trial Team. It was done on week 2, week 4, and week 6 after operations. Subjects were thoroughly examined by medical specialists or medical officers who are part of the Clinical Trial Team at every visit.

### 3.5. Sample Size Determination

The sample sizes are calculated based on two means formula (using G Power software), the power of the study taken at 90% and alpha (type one error) as 0.5%. The calculations are based on previous study [2],

  Power = 90%
  Type 1 error (*α*) = 0.5%
  SD = 14mm
  Expected detectable of mean difference between group = 10mm
  The sample size required for both study limb = 92
  Assuming 10% dropped out rate = 10
  Total number participants required for the study = 102
  Computer calculation
  F tests-ANOVA: repeated measures, between factors
  Actual power = 0.901176

## 4. Results

### 4.1. Description of Demographic Data

The demographic data of our study patient were summarized in Table 2. Out of 102 patients, only 81 patients completed follow-up and were analyzed. The mean age was 39 years (SD 8.89) for Snakehead fish (*Channa striatus)* spray and 41 years (SD 8.95) for placebo. 63 (77.7%) were male patient. Majority of the patient were Malay ethnic (98.8%).

There are 3 types of surgery involved in this study which are hernioplasty, excision biopsy, and thyroidectomy. Majority of our patients undergo hernioplasty (n 61, 76.5%), while 17.2% undergo excision biopsy (n=14) and 6.1% undergo thyroidectomy (n=5).

### 4.2. Comparison of Visual Analog Pain Score (VAPS) between Snakehead Fish (*Channa striatus)* Spray and Placebo Based on Time

Comparison of Visual Analog Pain Score (VAPS) between Snakehead fish (*Channa striatus)* spray and Placebo based on time is measured by repeated measure ANOVA shown in Table 3. There was significant difference of patient's pain and score based on VAPS between Snakehead fish (*Channa striatus)* spray and placebo (F-stat (df) = 4.80 (2), p-value = 0.010)

Estimated marginal means of visual analog score on weeks 2, 4, and 6 were plotted and it shows significant difference between Snakehead fish (*Channa striatus)* spray (mean=0.80, CI 0.61-0.99) and placebo (mean 1.26, CI 1.07-1.45) (Figure 2).

### 4.3. Comparison of Visual Analog Cosmetic Score (VACS) between Snakehead Fish (*Channa striatus)* Spray and Placebo Based on Time

The change in visual analog score between the initial, mid, and final follow-up attempted was analyzed using repeated measures ANOVA controlling for sample (placebo or trial); the interaction between both groups was significant: F-stat (df) = 2.68 (2), p-value <0.001 (Table 4). Mean parameter estimates are shown in Figure 3. Improvement of the score increased as the time of follow-up increased from 2^nd^ to 4^th^ to 6^th^ week. There was significant difference in estimated improvement between 4^th^ and 6^th^ week of follow-up completed. Figure 3 shows the estimated marginal means for the first mid and final follow-up score.

### 4.4. Comparison of Vancouver Scar Scale (VSS) between Snakehead Fish (*Channa striatus)* Spray and Placebo Based on Time

According to Vancouver Scar Scale, the worse cosmetic outcome score is 13. In our study, we found the mean score for Snakehead fish (*Channa striatus)* spray in week 2 is 3.97 (CI 3.39-4.56) which is lower compared to placebo 4.35, (CI 3.81-4.89). Overall, there was significant difference of mean resultant scars based on VSS between Snakehead fish (*Channa striatus)* spray and placebo (F-stat (df) = 1.72 (2), p-value = 0.011) as in Table 5 and Figure 4.

### 4.5. Comparison of Wound Evaluation Scale (WES) between Snakehead Fish (*Channa striatus)* Spray and Placebo Based on Time

There was significant difference of mean wound healing based on WES between Snakehead fish (*Channa striatus)* spray and placebo (F-stat (df) = 3.09 (2), p-value = 0.048) as in Table 6 and Figure 5.

## 5. Discussion

Snakehead fish (*Channa striatus)* has been used in traditional wound healing remedy for decades ago until today. It has been utilized not only in Malaysia, but also in most of country in South East Asia. In Malaysia, Malays believe that eating Snakehead fish (*Channa striatus)* during postdelivery period will enhance the recovery of the wound.

The effectiveness of topical application of Snakehead fish (*Channa striatus)* cream has been reported before. It shows that it does enhance wound healing by increasing tensile strength and increases fibroblast count and hydroxyproline level [9]. However, there is no article that describes the effectiveness of topical Snakehead fish (*Channa striatus)* spray on human. Thus, we decided to conduct a clinical trial on the effectiveness of Snakehead fish (*Channa striatus)* extract spray on clean surgical wound and we observed its pain control and cosmetic outcome.

In our study, we recruited 102 patients randomized into two group: treatment group A (n=51) and group B placebo (n=51). Patient was subsequently followed up on second, fourth, and sixth week in our surgical clinic for photographic and pain score assessment. We utilize the local product of Snakehead fish (*Channa striatus)* extract spray in our study. It has been manufactured by Skin Fix Company. Meanwhile we used Opsite spray as a placebo. Both has transparent and odorless spray droplet. Twenty-one patients were excluded due to not compliance to medication and defaulted follow-up. We then evaluated remaining 81 patients who completed follow-up on the effect of Snakehead fish (*Channa striatus)* extract spray on clean wound particularly pain and cosmetic effect postoperatively. Group A, the treatment group (n=41), and group B, the placebo group (n=40), receive Opsite spray.

For pain assessment we use Visual Analog Pain Score (VAPS). In our study we have proved that topical application of Snakehead fish (*Channa striatus)* extract does improve local analgesic effect in second, fourth, and sixth week postoperative period. The patients treated with Snakehead fish (*Channa striatus)* spray display a better outcome in terms of pain control compared to placebo. During analysis using repeated measure ANOVA, there was significant difference of patient's pain score based on VAPS between Snakehead fish (*Channa striatus)* spray and placebo (F-stat(df) = 4.80 (2), p-value = 0.010). Initial mean of visual analog score on week 2 shows significant difference between Snakehead fish (*Channa striatus)* spray (mean=0.80, CI 0.61-0.99) and placebo (mean 1.26, CI 1.07-1.45).

**Figure 1:**
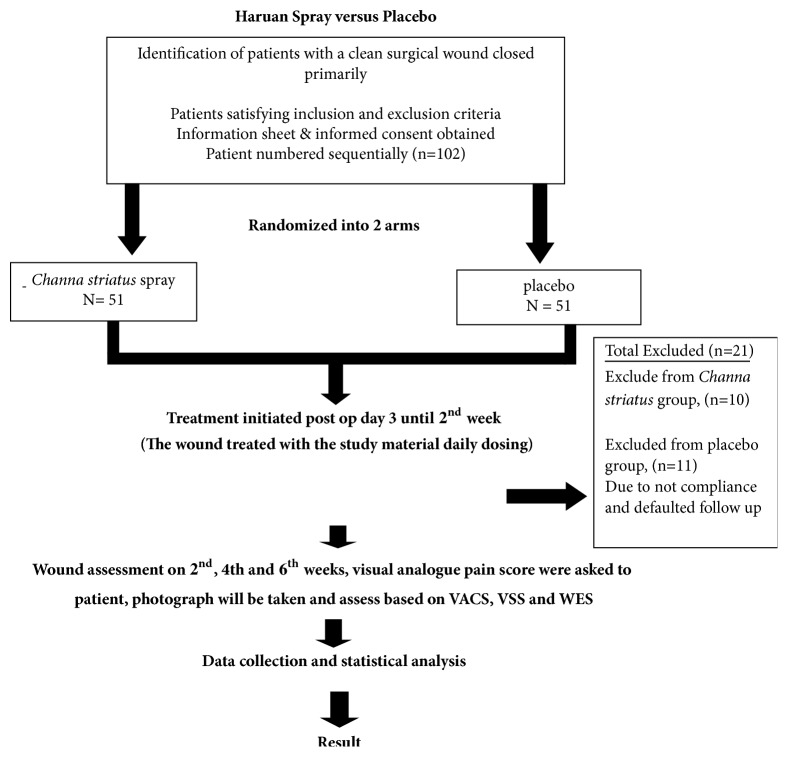
Flowchart of the study.

**Figure 2:**
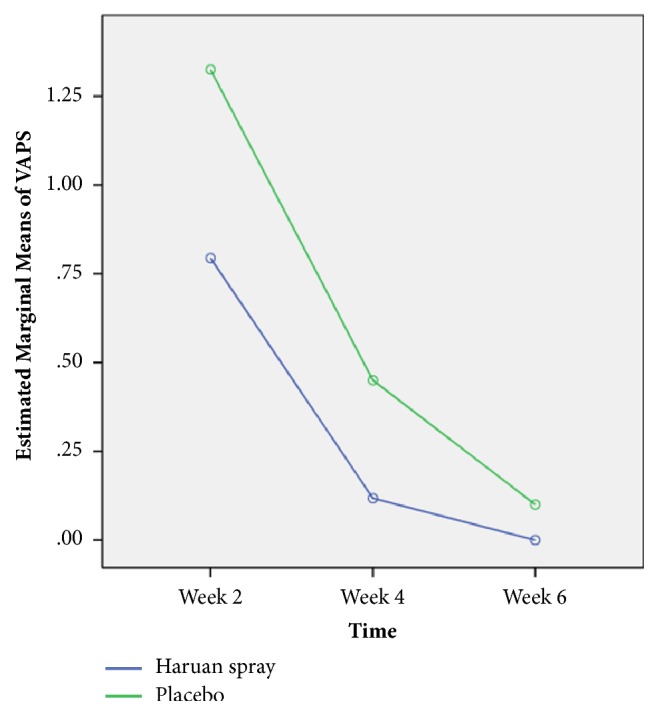
Comparison of estimated mean (estimated marginal means) of VASP for week 2, week 4, and week 6 interventions between Snakehead fish (*Channa striatus)* spray and placebo by comparing repeated measures ANOVA (n=81).

**Figure 3:**
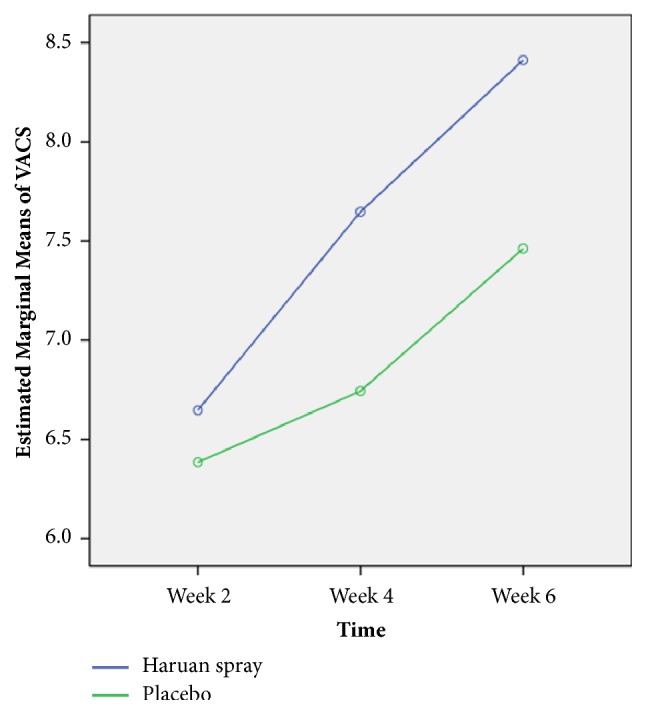
Comparison of estimated mean (estimated marginal means) of VACS for week 2, week 4, and week 6 interventions between Snakehead fish (*Channa striatus)* spray and placebo by comparing repeated measures ANOVA (n=81).

**Figure 4:**
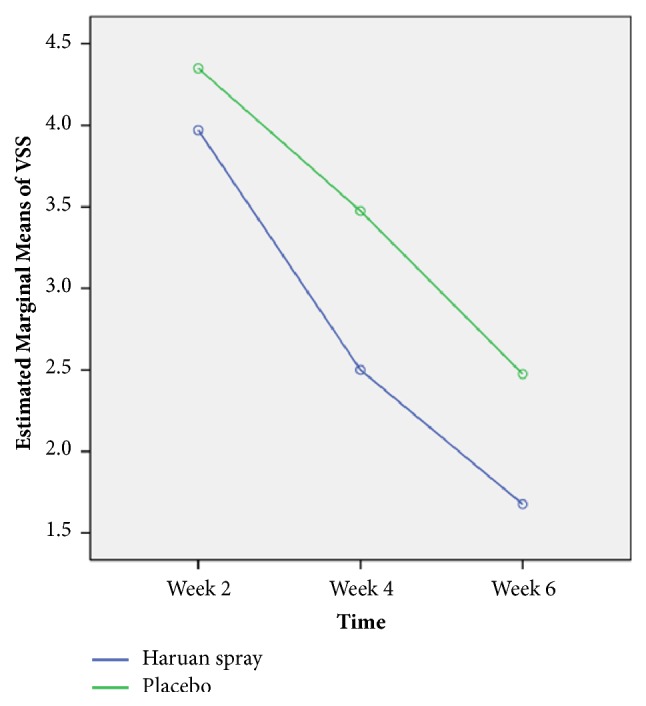
Comparison of estimated mean (estimated marginal means) of VSS for week 2, week 4, and week 6 interventions between Snakehead fish (*Channa striatus)* spray and placebo by comparing repeated measures ANOVA (n=81).

**Figure 5:**
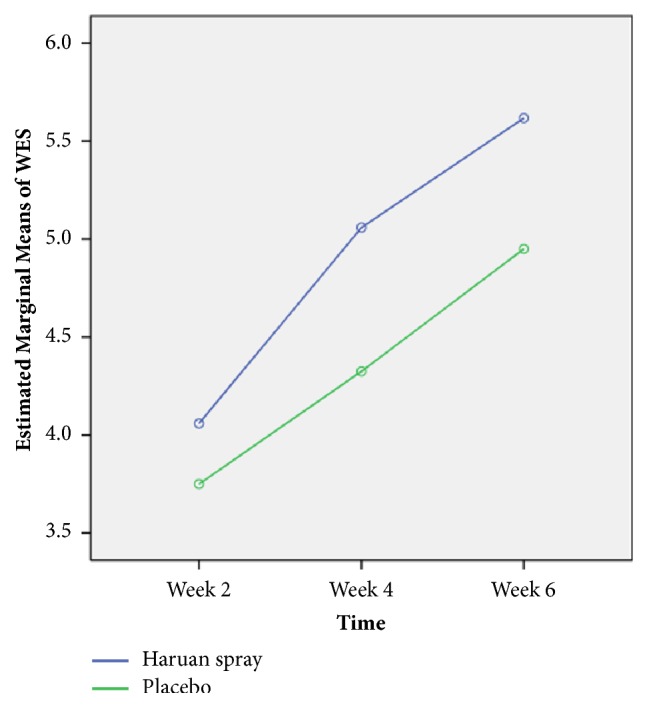
Comparison of estimated mean (estimated marginal means) of WES for week 2, week 4, and week 6 interventions between Snakehead fish (*Channa striatus)* spray and placebo by comparing repeated measures ANOVA (n=81).

This result also shows similar mean pattern for the week 4 and week 6 as shown in Figure 1. This is consistent with previous study [11, 13]. The proposed mechanism is that lipoamino acid and n-arachidonoyl glycine suppress the pain sensation by modulating the pain transmitter in the synaptic cleft [14, 15]. It was believed that extracts also enhance the activity of morphine [11, 15]. Apart from that, high concentration of arachidonic acid in Snakehead fish (*Channa striatus) *extract which also has functions in the antinociceptive pathways, was found in many studies [16, 17]. Previous study shows that application of Snakehead fish (*Channa striatus) *in post-C-section patient shows that it improves the pain control outcome [18].

In terms of cosmetic outcome, there are various methods of assessing the wound. In our study we used Visual Analog Cosmetic Scale (VACS), Wound Evaluation Scale (WES), and Vancouver Scar Scale (VSS) which have been validated in previous study [19, 20]. In our study, we found that the cosmetic outcome shows a consistent significant better cosmetic result in Snakehead fish (*Channa striatus)* spray group for all the 3-scoring system which is VACS (F-stat(df) = 2.68 (2), p-value <0.001), WES (F-stat(df) = 3.09 (2), p-value = 0.048), and VSS (F-stat(df) = 1.72 (2), p-value = 0.011). In our study also we observe a consistent result between WES and VAS as both show similar result pattern with minimal clinical important different (MIDC) as was describe in previous study [19]

Many studies have been published regarding the specific element of Snakehead fish (*Channa striatus)* especially its meat and roe. The high content of essential amino acid and fatty acid is the main factor that contributes to speedy recovery of the wound [10, 16, 21]. These two components are reported to promote wound healing. It initiates collagen synthesis and reepithelialization in the healing wound.* Arginine *is also one of the potent amino acids that promote wound healing [22]. The polyunsaturated fatty acid (PUFA) has an important role in immune respond in healing process [23, 24]. It is an important component of cell plasma membrane synthesis (biphospholipid layer) [25]. PUFA is also an important substrate of production of* prostaglandin, thromboxane, leukotrienes,* and* lipoxin* synthesis [25]. Deficiency of this component will slow down the healing process [17].

We have 1 case of hypertrophic scar in Snakehead fish (*Channa striatus)* spray group, and none was seen in placebo group. However there is no case of keloid seen in either group. Hypertrophic scars are define as raised fibrous connective tissue in the dermis and adjacent subcutaneous tissue after traumatic or burn wound healing [26]. It is due to excessive accumulation of scar collagen and presence of abundance myofibroblast cell, a contractile cell [6]. Few studies have shown that* Channa striatus* application to the wound increases the tensile strength [1, 5].

Out of 81 patients, none was found to have surgical site infection (SSI). The rate for SSI in previous study is up to 2.1% [27]. Although this study did not specifically look at this issue, it shows that application of Snakehead fish (*Channa striatus)* extract did not increase the risk to get surgical site infection. This is postulated due to the antimicrobial effect of Snakehead fish (*Channa striatus)* extract.

As part of the wound healing process, antimicrobial activity is equally important. The antimicrobial properties have been studied by CARE research team. The investigation showed a broad spectrum of antibacterial activity of skin mucus against* Aeromonas hydrophila, Pseudomonas aeruginosa,* and* Vibrio anguillarum* and intestinal mucus against* A. hydrophila* [11]

The present study has several limitations. Difference in long-term effect of Snakehead fish (*Channa striatus)* extract spray on clean wound is not investigated. Longer clinical trials involving more patients are warranted. This is not single surgeon based study; therefore there is experience bias in terms of operating skill. Some patient defaulted after discharge from ward and outcome cannot be assessed.

Since our study once again proves that Snakehead fish (*Channa striatus)* extract* (Channa striata)* spray has shown a significant better pain score result and cosmetic outcome on clean wound, it has opened a window of opportunity to study the long term outcome. The future study on the use of Snakehead fish (*Channa striatus)* extract spray can also be extended on the clean-contaminated or contaminated wound. The other potential study is the effect of Snakehead fish (*Channa striatus)* pill and topical application of Snakehead fish (*Channa striatus)* cream as a dressing and its effect on wound healing.

## 6. Conclusion

In current study, it is clearly demonstrated that application of Snakehead fish (*Channa striatus)* extract* spray* on clean wound has shown a significant better pain score result and cosmetic outcome on week 2, week 4, and week 6 comparatively with placebo. It was not associated with additional morbidity in terms of its cosmetic outcome postoperatively at second, forth, and sixth week.

## Figures and Tables

**Table 1:** Composition of amino acids and fatty acid in *Channa striatus* extract.

	Fillet	Roe	Mucus
Amino acids	Glycine		
	Glutamic acid	(No study)	(No study)
	Arginine		
	Aspartic acid		

Fatty acids	Eicosapentaenoic	Eicosapentaenoic	Oleic acid
	Acid (EPA)	Acid (EPA)	Linoleic acid
	Docosahexaenoic	Docosahexaenoic	
	Acid (DHA)	Acid (DHA)	
	Palmitic acid	Hexadecanoid acid	
	Oleic acid	Oleic acid	
	Stearic acid	Linoleic acid	
	Arachidonic acid		

Adopted from [12].

**Table 2:** Demographic distribution (n=81).

	Haruan Spray (n=41)	Placebo (n=40)
Variable	Frequency (%)	
Gender		
Male	30 (73.2)	33 (82.5)
Female	11 (26.8)	7 (17.5)
Age (years)*∗*	39.4 (8.89)	41.2 (8.95)
Race		
Malay	40 (97.6)	40 (100.0)
Chinese	1 (2.4)	0 (0.0)
Type of surgery		
Hernioplasty	29 (70.7)	33 (82.5)
Excision biopsy	9 (22.0)	5 (12.5)
Thyroidectomy	3 (7.3)	2 (5.0)

**Table 3:** Comparison of VAPS between Snakehead fish (*Channa striatus)* spray and Placebo based on time (n=81).

Time	Group	Mean	95% Confidence Interval	
Week 2	*Channa striatus* spray	0.79	0.54	1.05
	Placebo	1.33	1.09	1.56
Week 4	*Channa striatus* spray	0.12	0.04	0.28
	Placebo	0.45	0.30	0.60
Week 6	*Channa striatus* spray	0.03	0.01	0.10
	Placebo	0.10	0.01	0.19

RM Anova: F-stat (df) = 4.80 (2), p-value = 0.010.

**Table 4:** Comparison of VACS between Snakehead fish (*Channa striatus)* spray and placebo based on time (n=81).

Time	Group	Mean	95% Confidence Interval	
Week 2	*Channa striatus* spray	6.65	6.28	7.02
	Placebo	6.39	6.04	6.73
Week 4	*Channa striatus* spray	7.65	7.29	8.00
	Placebo	6.74	6.41	7.08
Week 6	*Channa striatus* spray	8.41	8.08	8.74
	Placebo	7.46	7.15	7.77

RM ANOVA: F-stat (df) = 2.68 (2), p-value <0.001.

Repeated measure ANOVA between group analyses with regard to time was applied.

**Table 5:** Comparison of VSS between Snakehead fish (*Channa striatus)* spray and placebo based on time (n=81).

Time	Group	Mean	95% Confidence Interval	
Week 2	*Channa striatus* spray	3.97	3.39	4.56
	Placebo	4.35	3.81	4.89
Week 4	*Channa striatus* spray	2.50	1.97	3.03
	Placebo	3.48	2.98	3.97
Week 6	*Channa striatus* spray	1.68	1.24	2.12
	Placebo	2.48	2.07	2.88

RM ANOVA: F-stat (df) = 1.72 (2), p-value = 0.011.

Repeated measure ANOVA between group analyses with regard to time was applied.

Assumptions of normality, homogeneity of variances, and compound symmetry were checked and fulfilled.

**Table 6:** Comparison of WES between Snakehead fish (*Channa striatus)* spray and Placebo based on time (n=81).

Time	Group	Mean	95% Confidence Interval	
Week 2	*Channa striatus *spray	4.06	3.68	4.44
	Placebo	3.75	3.40	4.10
Week 4	*Channa striatus *spray	5.06	4.78	5.33
	Placebo	4.33	4.07	4.58
Week 6	*Channa striatus *spray	5.62	5.38	5.86
	Placebo	4.95	4.73	5.17

RM ANOVA: F-stat (df) = 3.09 (2), p-value = 0.048.

Repeated measures ANOVA between group analyses with regard to time was applied. Assumptions of normality, homogeneity of variances, and compound symmetry were checked and fulfilled.

## Data Availability

The data used to support the findings of this study are available from the corresponding author upon request.
